# Effective Exon Skipping and Dystrophin Restoration by 2′-O-Methoxyethyl Antisense Oligonucleotide in Dystrophin-Deficient Mice

**DOI:** 10.1371/journal.pone.0061584

**Published:** 2013-04-26

**Authors:** Lu Yang, Hongjing Niu, Xianjun Gao, Qingsong Wang, Gang Han, Limin Cao, Chunquan Cai, Jan Weiler, Haifang Yin

**Affiliations:** 1 Research Centre of Basic Medical Science, Tianjin Medical University, Heping District, Tianjin, China; 2 Novartis Institutes for BioMedical Research Inc., NIBR Biologics Center, Cambridge, Massachusetts, United States of America; Goethe University, Germany

## Abstract

Antisense oligonucleotide (AO)–mediated exon-skipping therapy is one of the most promising therapeutic strategies for Duchenne Muscular Dystrophy (DMD) and several AO chemistries have been rigorously investigated. In this report, we focused on the effect of 2′-O-methoxyethyl oligonucleotides (MOE) on exon skipping in cultured *mdx* myoblasts and mice. Efficient dose-dependent skipping of targeted exon 23 was achieved in myoblasts with MOE AOs of different lengths and backbone chemistries. Furthermore, we established that 25-mer MOE phosphorothioate (PS) AOs provided the greatest exon-skipping efficacy. When compared with 2′O methyl phosphorothioate (2′OmePS) AOs, 25-mer MOE (PS) AOs also showed higher exon-skipping activity *in vitro* and in *mdx* mice after intramuscular injections. Characterization of uptake *in vitro* corroborated with exon-skipping results, suggesting that increased uptake of 25-mer MOE PS AOs might partly contribute to the difference in exon-skipping activity observed *in vitro* and in *mdx* mice. Our findings demonstrate the substantial potential for MOE PS AOs as an alternative option for the treatment of DMD.

## Introduction

Duchenne muscular dystrophy (DMD) is a lethal muscle degenerative disease that arises from mutations, typically large deletions, in the DMD gene resulting in out-of-frame dystrophin transcripts and ultimately in the lack of functional dystrophin protein. Antisense oligonucleotides (AOs) are short single-stranded nucleic acids capable of effecting splice correction of aberrant disease-related pre-mRNA transcripts in order to restore their function [1]. Such AOs have been shown to correct aberrant out-of-frame dystrophin transcripts via the exclusion of specific dystrophin exons, thereby restoring the open reading frame to generate a shortened but functional dystrophin protein product [2].

Exploitation of AOs as splice correcting therapeutic agents for DMD was successfully demonstrated in mdx mice and DMD patient cells [3], [4], [5]. Recently, AO-mediated exon-skipping strategy for DMD has progressed into clinical trials in the UK and the Netherlands with some promising results [6], [7], [8], [9]. However, systemic restoration of dystrophin expression in vivo will be important for therapeutic correction in DMD patients and this has proven considerably more challenging in animal models with currently tested AO chemistries (i.e. 2′OmePS, 2′-O-methyl phosphorothioate RNA; PMO, phosphorodiamidate morpholino and PNA, peptide nucleic acid) as previously reported [10], [11], [12], [13], [14], [15], though the former two AO chemistries are currently in phase IIa/IIb clinical trials. Low level of systemic dystrophin restoration is attributed to poor delivery efficiency of current AOs, which was supported by recent reports on cell-penetrating peptides (CPPs) modified PMO from our group and others [16], [17], [18]. By conjugating CPPs to PMO, the exon-skipping efficacy and level of dystrophin expression can be significantly enhanced [19], [20], however the reported toxicity profiles of CPPs may limit their clinical use.

Nevertheless, other AO chemistries may be more amenable to cellular uptake *in vivo* and thus improve exon-skipping efficiency. Notable amongst these are 2′-*O*-methoxyethyl phosphorothioate RNA (MOE PS) AOs. MOEs are RNA analogues formed by modifying the 2′ position in the ribose sugar with the methoxyethyl group and by replacing the phosphodiester bond of the ribose backbone with a phosphorothioate bond, which is stable and resistant to nucleases and imparts high binding affinity and sequence specificity [21]. MOEs have been successfully used to down-regulate various targeted mRNAs via an RNase H-dependant pathway in the form of MOE-DNA gapmers [22], [23], [24], [25], [26], [27], and furthermore it has shown potential in mediating splicing in other models [28], [29], whereas their potential in mediating splice correction in DMD remains to be exploited.

Here, we investigated the potential of MOE AOs as splice correcting therapeutic agents for DMD by comparing the exon-skipping efficiency of MOE AOs of different length in cultured *mdx* myoblast and their exon-skipping activity in *mdx* mice with 2′OMePS AOs. We demonstrated that MOE (PS) AOs can effectively induce exon-skipping better than 2′OMePS AOs both *in vitro* and in *mdx* mice and that the increased exon-skipping efficiency is probably due to increased cellular uptake.

## Materials and Methods

### Animals

Six to 8-week old *mdx* mice were used in all experiments (3 mice in the test and control groups). The experiments were carried out in the animal unit, Tianjin Medical University (Tianjin, China) according to procedures authorized by the institutional ethical committee (Permit Number: SYXK 2009-0001). Mice were killed by cervical dislocation at desired time points, and muscles and other tissues were snap-frozen in dry ice-cooled isopentane and stored at −80°C.

### Oligonucleotides

Three MOE AOs with different lengths and backbones were used in this study. Details of tested AOs were shown in Table 1. All AOs were synthesized as described previously [30]. Different MOE AO lengths and positions with respect to boundary region of exon and intron 23 of murine *DMD* gene were identical to the ones reported previously [14].

**Table 1:** Oligonucleotide nomenclature and sequence.

Name	Sequence	Abbreviation	Length
2′-O-Methyl-phosphorothioate RNA	5′-GGCCAAACCUCGGCUUACCU-3′	2′OmePS	20
Murine_exon_23 M-2+18D	5′-GGCCAAACCTCGGCTTACCT-3′	MOE20(PS)	20
M23D(+7−18)_moe_PO	5′-GGCCAAACCTCGGCTTACCTGAAAT-3′	MOE25(PO)	25
M23D(+7−18)_moe_PS	5′-GGCCAAACCTCGGCTTACCTGAAAT-3′	MOE25(PS)	25

### Cell culture and transfection

H_2_K *mdx* myoblasts [31] were cultured at 33°C in 10% CO_2_ in Dulbecco's modified Eagle's medium (DMEM) supplemented with 20% fetal calf serum, 2% chicken embryo extract (PAA Laboratories Ltd, Yeovil, UK), and 20 U/ml γ-interferon (Roche, Herts, UK). Cells were then treated with trypsin and plated at 5×10^4^ cells per well in 24-well plates coated with 200 µg/ml gelatin. H_2_K *mdx* cells were transfected 24 h after trypsin treatment in a final volume of 0.5 ml of antibiotic- and serum-free Opti-MEM. The weight ratio of tested AOs and lipofectin (Invitrogen) was 1∶2.5 according to the instructions provided by the supplier. After 5 h of incubation, the transfection medium was replaced with DMEM.

### Exon skipping in *mdx* mouse myotubes

Myotubes were obtained from confluent H2K *mdx* cells seeded in gelatin coated 24-well plates following 2 days of serum deprivation (DMEM with 5% horse serum). 500 nM MOEs and 2′Ome PS AOs were incubated with myotubes for 4 h in 0.5 ml OptiMEM and then replaced by 1 ml of DMEM/5% horse serum media for further incubation. After 48 h myotubes were washed twice with PBS and total RNA was extracted with 0.5 ml of TRI Reagent (Sigma, UK).

### RNA extraction and nested reverse transcriptase-polymerase chain reaction (RT-PCR) analysis

Total RNA was extracted from transfected cells with Trizol and 200 ng of RNA template was used for 10 µl RT-PCR with OneStep RT-PCR kit. The primer sequences for initial RT-PCR were Exon20Fo 5′-CAGAATTCTGCCAATTGCTGAG-3′ and Exon26Ro 5′-TTCTTCAGCTTTTGTGTCATCC-3′ for reverse transcription from mRNA and amplification of cDNA from exons 20–26. The primer sequences for the second round were Exon20F1 5′-CCCAGTCTACCACCCTATCAGAGC-3′ and Exon24R1 5′-CCTGCCTTTAAGGCTTCCTT-3′. The cycle conditions were as previously described [15]. The products were examined by electrophoresis on a 2% agarose gel.

### Cell toxicity assay

A modified WST-8 kit, which measures the metabolic activity of viable cells [26], was used to evaluate the toxicity of three MOE AOs in H_2_K *mdx* cells at 1, 5 and 10 µM concentrations. Cells were plated at 5×10^3^ cells per well in 96-well plates overnight and treated with different AOs compared with untreated cells as a control for 24 h, and then incubated with 10 µl WST-8 for 4 h. During this incubation period, viable cells convert WST-8 to a water-soluble formazan dye, which is then quantified using an enzyme-linked immunosorbent assay by a plate reader with an absorbance value at 450 nm.

### Immunohistochemistry

8 µm sections were cut from at least two-thirds of muscles at 100 µm intervals. The sections were then examined for dystrophin expression with a polyclonal rabbit antibody against the dystrophin carboxyl terminal region (ab15277, Abcam, UK). Polyclonal antibodies were detected by goat-anti-rabbit immunoglobin G Alexa Fluro 594 (Molecular probe, Invitrogen, UK). The maximum number of dystrophin-positive fibres in one section was counted and muscle fibres were defined as dystrophin- positive when more than two-thirds of the single fibre showed continuous staining.

### Protein extraction and Western blot

Protein extraction and Western blot were carried out as previously described [17] Various amounts of protein from wild-type *C57BL6* mice were used as positive controls and corresponding amounts of protein from muscles of treated or untreated *mdx* mice were loaded onto sodium dodecyl sulphate-polyacrylamide gel electrophoresis gels (4% stacking, 6% resolving). The membrane was then washed and blocked with 5% skimmed milk and probed overnight with DYS1 (Abcam, UK) for the detection of dystrophin protein and α-actinin (Sigma, US) as a loading control. The bound primary antibody was detected by horseradish peroxidise-conjugated goat anti-mouse immunoglobin G (Sigma, US) and the ECL western blot analysis system (Millipore, US). The intensity of the bands obtained from treated *mdx* muscles was measured by Image J software.

### Statistical analysis

All data are reported as mean values±SEM. Statistical differences between treated groups and control groups were evaluated by SigmaStat (Systat Software, UK) and the Student's *t*-test.

## Results

### MOE AOs induce effective dystrophin exon skipping *in vitro* in a dose-dependent manner

**Figure 1:**
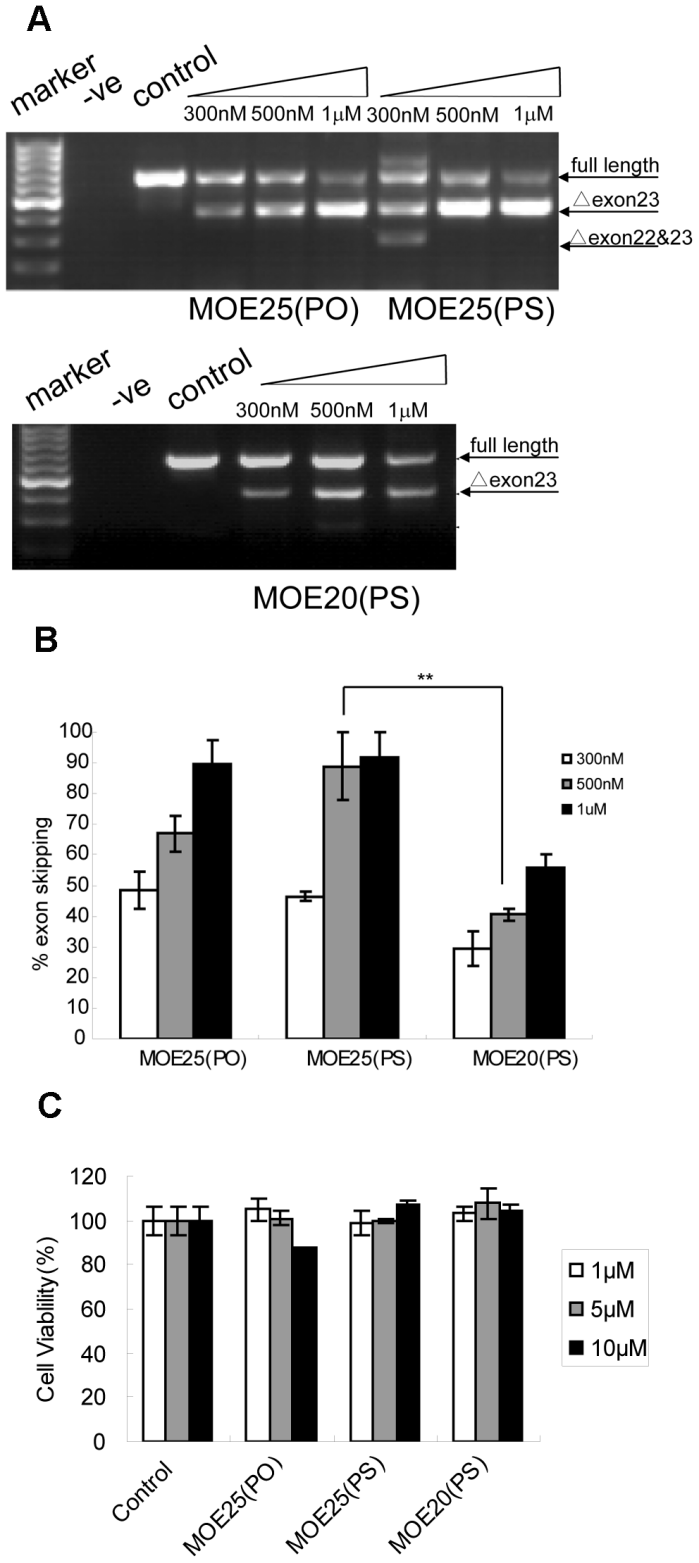
Dose-dependent analysis for different MOE AOs in H_2_K *mdx* cells. (A) RT-PCR results for different MOE AOs in H_2_K *mdx* cells at different concentrations from 300 nM to1 µM. ΔExon 23 represents exon 23 skipped PCR product; ΔExon 22&23 indicates both exon 22 and exon 23 skipped PCR product. (B) Quantification of percentage of exon 23 skipping for different MOE AOs at different concentrations. The data show higher activity with MOE25(PS) than that of MOE20(PS) AOs at a concentration of 500 nM (n = 6, **p<0.001). (C) A WST-8 cytotoxicity assay for tested AOs in H_2_K *mdx* cells with AO concentrations up to 10 µM.

In order to evaluate the ability of MOE AOs to induce exon-skipping of the dystrophin mRNA, we transfected H_2_K *mdx* cells derived from *mdx* mouse with 20-mer and 25-mer MOE AOs directed against different sequences at the exon-intron boundary of exon and intron 23 of the murine *DMD* gene as reported previously (14) (shown in Table 1). As the replacement of phosphodiester bond (PO) with phosphorothioate bond (PS) in MOE chemistry increases stability and sequence-specificity, we wished to compare the exon-skipping activity of identical MOE sequences with PO and PS backbones in cell culture side-by-side. Different concentrations of MOE25(PS) and MOE25(PO) were tested in H_2_K *mdx* cells. RT-PCR results showed clear exon 23 exclusion in cells treated with MOE AOs at a concentration of 300 nM at 48 h post-transfection, which is the peak time-point for exon-skipping activity of other AO chemistries *in vitro* as shown in our previous study [27]. Concentration-dependent exon-skipping was evident for MOE25 (PO) AOs with exon-skipping of 95% of transcripts at 1 µM concentration. Whereas for MOE25(PS), about 95% exon-skipping was achieved at 500 nM and no significant difference was detected between 500 nM and 1 µM, suggesting that MOE25(PS) AOs reached saturation at the concentration of 500 nM (Fig.1A–B).

Furthermore, as it was reported that 25-mer 2′OmePS AOs induced less effective exon-skipping than that of 20-mer ones [32], so we wished to examine whether the case is the same for MOE (PS) AOs. Different concentrations of MOE25(PS) and MOE20(PS) were tested in H_2_K *mdx* cells at 48 h after transfection and RT-PCR analysis revealed that MOE25(PS) AOs induced significantly higher exon skipping efficiency than MOE20(PS) at each tested concentration, indicating 25-mer is more effective than shorter ones.

We next examined the possible toxicity of three MOE AOs in H_2_K *mdx* cells using a WST-8 assay, which measures the metabolic activity of viable cells [15]. Cells were plated in 96-well microplates overnight and treated with different concentrations of MOE25(PS), MOE20(PS) and MOE25(PO) AOs for 12 h in the absence of lipofectine, and then incubated with WST-8 for about 4 h. Toxicity or cell proliferation inhibition was not observed when cells were treated with MOE25(PS) and MOE20(PS) AOs at concentrations ranging from 1 µM to 10 µM, the highest of which is 10-fold higher than concentrations used for cell transfection experiments (Fig. 1C).

### Time-course studies for MOE AOs *in vitro*

**Figure 2:**
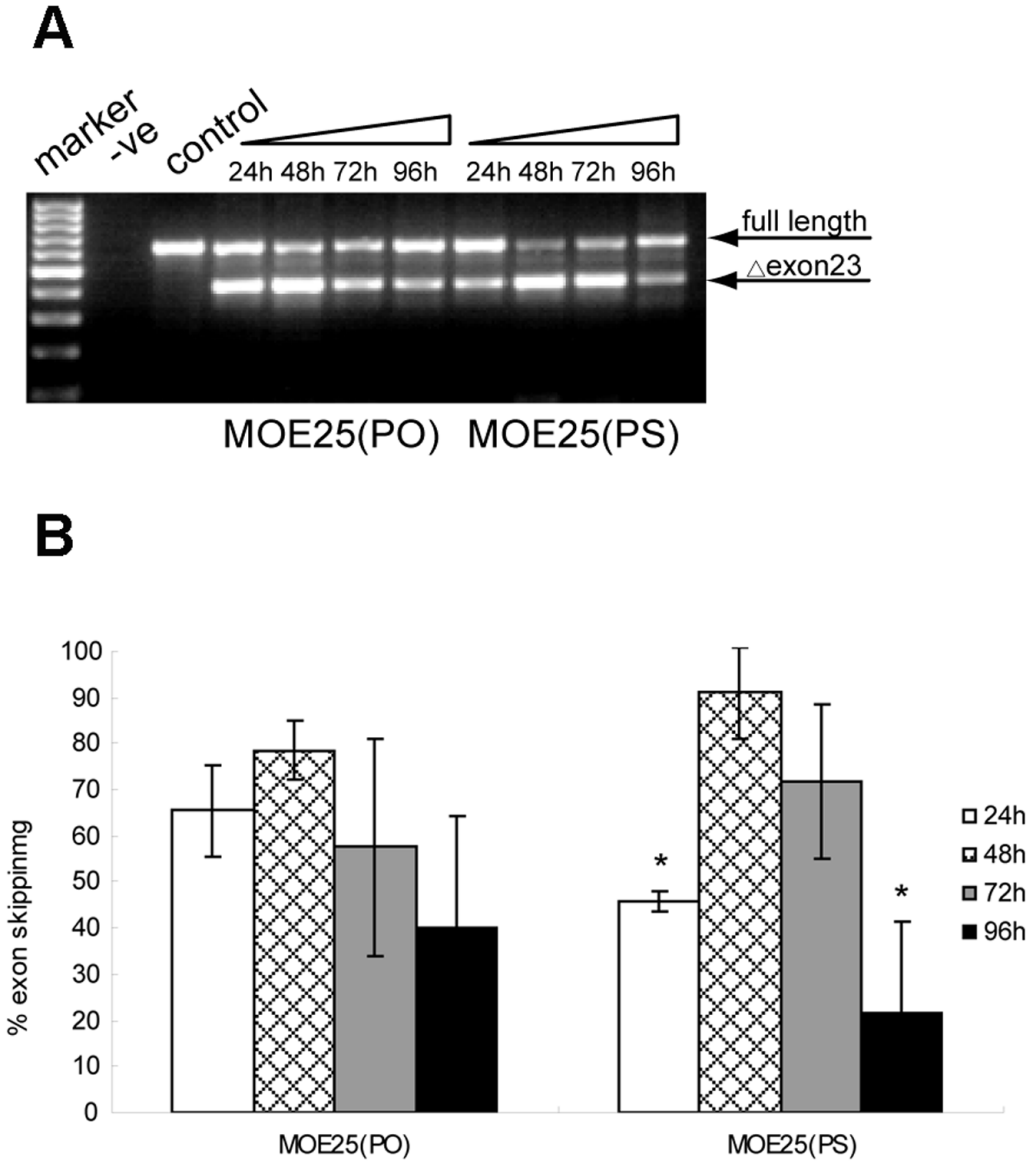
Time-course analysis for different MOE AOs in H_2_K *mdx* cells. (A) RT-PCR results for MOE25(PS) and MOE25(PO) AOs in H_2_K *mdx* cells at different time-points from 24 to 96 h after transfection. (B) Quantification of percentage of exon 23 skipping for MOE25(PS) and MOE25(PO) AOs at different time-points. The data indicate that MOE25(PS) induced significantly higher percentage of exon 23 skipping at 48 h than those of other time-points, with the exception of 72 h time-point (*p<0.05).

To define the optimal time-point for detecting exon-skipping activity of MOE AOs *in vitro*, we transfected MOE25(PS) and MOE25(PO) into H_2_K *mdx* cells at the concentration of 500 nM as the exon skipping activity for MOE25(PS) reached a plateau at this concentration (Fig.2A). A series of time-points were evaluated and RT-PCR results showed that 48 h post-transfection is the peak time for measuring exon-skipping with significantly higher exon23 skipping efficiency than those of other time-points, with the exception of 72 h time-point as shown in Fig. 2B. MOE25(PS) and MOE25(PO) AOs demonstrated a similar exon-skipping pattern with the highest exon skipping efficiency achieved at 48 h post-transfection and lower level of exon-skipping detected at 96 h after transfection.

### Direct comparison of *in vitro* exon skipping activities of MOE and 2′OmePS AOs

**Figure 3:**
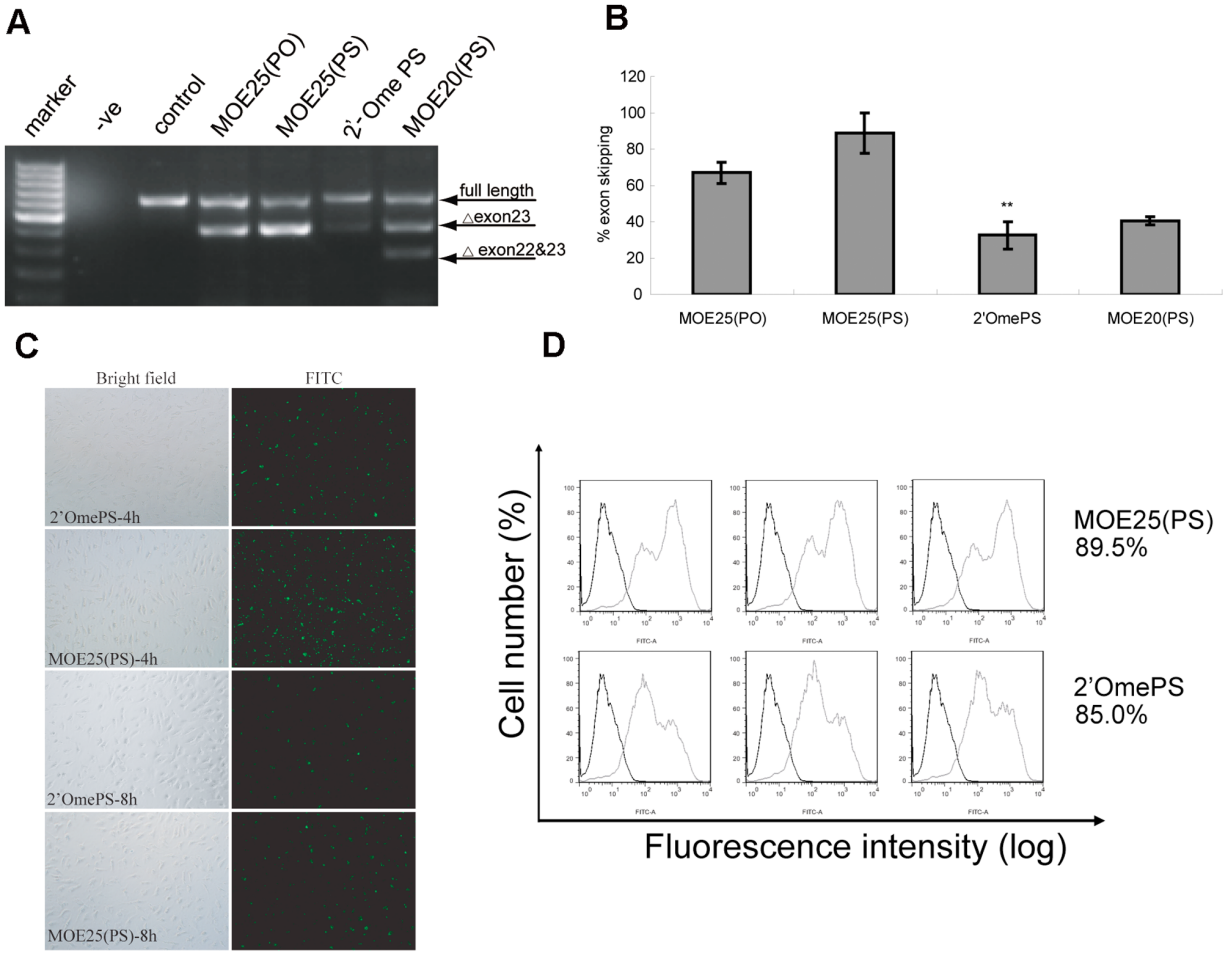
Direct comparison between MOE and 2′OmePS AOs in inducing exon skipping in H_2_K *mdx* cells. (A) RT-PCR results for 500 nM MOE and 2′OmePS AOs in H_2_K *mdx* cells at 48 h after transfection. (B) Quantification of percentage of exon 23 skipping for MOE and 2′OmePS AOs at 48 h after transfection. The data indicate significant increased exon skipping was detected in cells treated with MOE25(PS) compared with 2′OmePS AOs (**p<0.001). (C) Cellular uptake of fluorescence-labeled MOE25(PS) and 2′OmePS AOs in H2K *mdx* cells at the concentration of 500 nM. The cellular uptake was monitored 4 h and 8 h post-transfection with fluorescence microscopy and the data indicate higher uptake observed at 4 h time-point for both AOs. (D) Quantative analysis of the transfection efficiency with flow cytometry. The data show much stronger fluorescence intensity observed in cells treated with MOE25(PS) AOs than those treated with 2′OmePS AOs.

MOE AOs are structurally similar to 2′OmePS AOs, which are currently being tested in clinical trials in the Netherlands. Now that we demonstrated that MOE AOs are effective in inducing exon-skipping in H_2_K *mdx* cells, therefore we wished to directly compare the exon-skipping activity of MOE AOs to that of 2′OmePS AOs *in vitro* and *in vivo*. The optimal concentration (500 nM) and time-point (48 h post-transfection) were utilized for the comparison study. RT-PCR results demonstrated that MOE25(PS) induced significantly higher exon-skipping than those of 2′OmePS and MOE20(PS), whereas a marginal increase was detected for MOE25(PS) compared to MOE25(PO) (Fig. 3A–B). These data were consistent with the results from dose-dependent and time-course studies, suggesting MOE25(PS) AOs are potential alternatives for exon-skipping in DMD. Moreover, we tested these AOs in differentiated H2K *mdx* myotubes and the results showed the same pattern as observed in undifferentiated H2K *mdx* myoblasts (Fig. S1), implying that exon skipping efficiency depends on sucessful delivery rather than state of cell differentiation.

Furthermore, we wished to understand whether the effective cellular uptake accounts for the efficient exon skipping activity with MOE25(PS) AOs observed in H2K *mdx* cells. To verify this possibility, we transfected fluorescence tagged MOE25(PS) AOs to H2K *mdx* cells at the concentration of 500 nM and monitored the cellular uptake with fluorescence microscopy at 4 h and 8 h post-transfection with lipofectine (Fig. 3C), followed by quantitative measurement with flow cytometry (FACS). The FACS results indicated that up to 90% transfection efficiency was achieved with MOE25(PS) AOs at 4 h after transfection. Compared with 2′Ome PS, much stronger intensity was observed in cells transfected with MOE25(PS) (Fig. 3D), suggesting more fluorescence-tagged MOE25(PS) AOs were taken up by H2K *mdx* cells. The cellular uptake results were consistent with the DMD exon skipping activity *in vitro*, implying the increased cellular uptake might partly contribute to the improved exon skipping activity observed with MOE25(PS) AOs.

### MOE25(PS) AOs induced effective exon skipping and dystrophin restoration in *mdx* mice by local intramuscular injection

**Figure 4:**
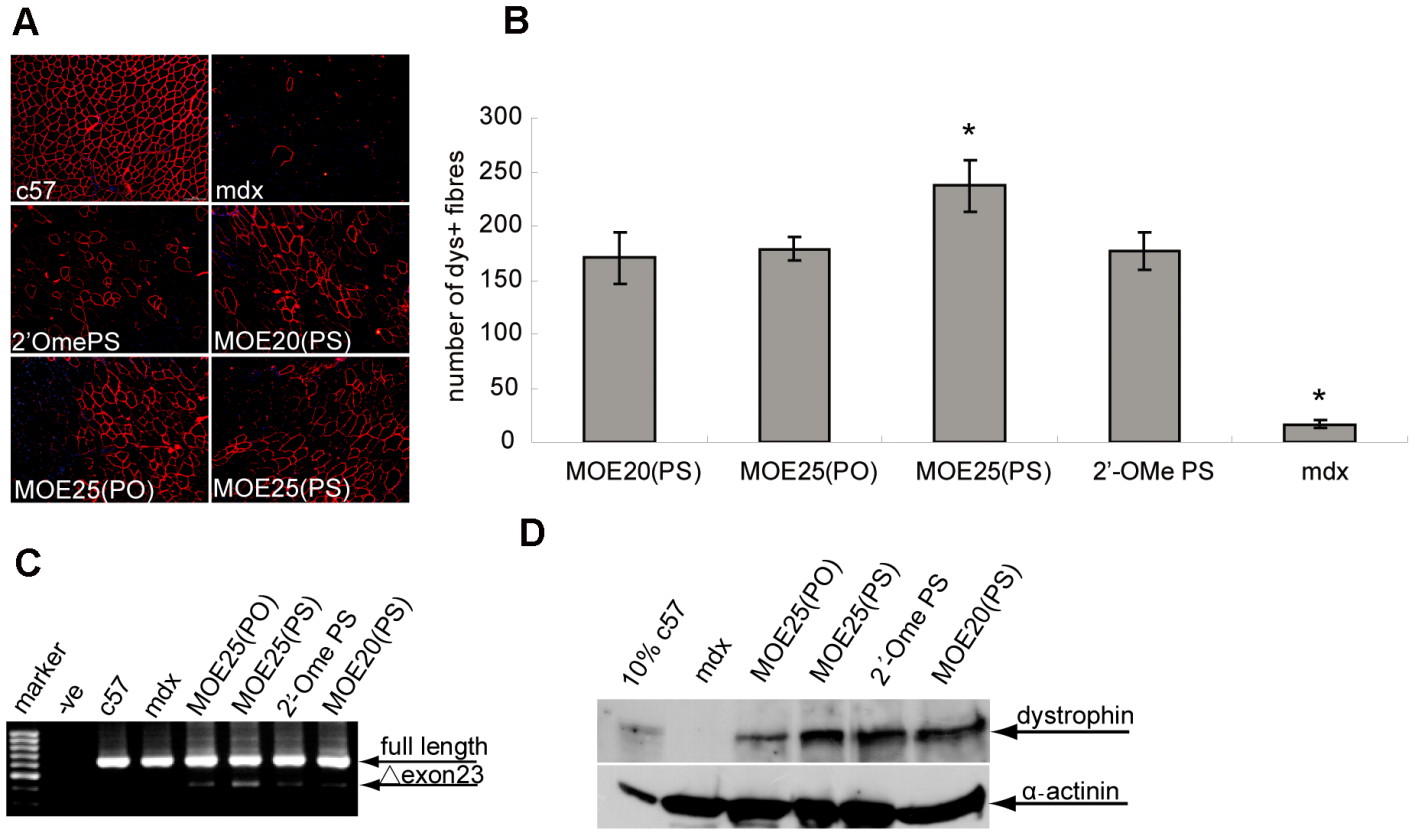
MOE AOs induce effective exon skipping and dystrophin restoration in *mdx* mice by local intramuscular injection. (A) Immunohistochemistry for dystrophin induction in TA muscles of adult *mdx* mice 2 weeks after one single intramuscular injection of 5 µg MOE25(PS), MOE25(PO), MOE20(PS) and 2′OmePS AOs (scale bar = 100 µm). (B) Quantitative evaluation of total dystrophin-positive fibres in TA muscles treated with MOE and 2′OmePS AOs at 2 weeks after a single injection. The data shows that MOE25(PS) AOs restored significantly higher number of dystrophin-positive fibres than those of other AOs and significant difference was observed for all tested AOs compared with untreated *mdx* control (*p<0.05). (C) RT-PCR to detect exon skipping efficiency at the RNA level demonstrated up to 10% exon 23 skpping in the TA muscle treated with MOE25(PS) AOs at 2 weeks after injection. This is shown by shorter exon skipped bands (indicated by Δexon23 for exon 23 skipping). (D) Western blot analysis for treated TA muscles at 2 weeks after one single intramuscular injection of MOE and 2′OmePS AOs. Total protein was extracted from TA muscles of adult *mdx* mice treated with different AOs and untreated control. Fifty microgram of total protein from untreated *mdx* mice TA muscles and treated muscle samples was loaded. Five microgram of total protein (10%) from *C57BL6* TA muscles was loaded as a normal control. No visible difference in the size of dystrophins between muscles treated with AOs and muscle from the normal *C57BL6* mouse. α-actinin was used as loading control.

To further examine the exon skipping activity of MOE AOs *in vivo*, we injected 5 µg of MOE25(PS), MOE25(PO), MOE20(PS) and 2′OmePS into tibialis anterior (TA) muscle of adult *mdx* mice, respectively. Treated TA muscles were harvested 2 weeks post-injection and assayed by immunohistochemistry. Immunohistochemical staining results revealed that substantial number of dystrophin-positive fibres were present in the injected region with uniform distribution throughout cross-sections in TA muscles treated by MOE25(PS) AOs (Fig. 4A). About 237±23 dystrophin-positive fibres were detected in TA muscles treated by MOE25(PS), which was significantly higher than those of other AOs and untreated, age-matched control *mdx* mice (Fig. 4B). In line with the immunostaining results, our RT-PCR data indicated that more effective exon 23 skipping was detected in samples treated with MOE25(PS) AOs than other AOs (Fig. 4C), whereas no difference was observed for MOE25(PO), MOE20(PS) and 2′OmePS. Western blot analysis further corroborated RT-PCR and immunostaining results showing up to 20% of normal level of dystrophin protein restored in TA muscles treated by MOE25(PS) AOs, while only about 10% and 15% of normal dystrophin protein levels were found in samples treated with MOE25(PO) and 2′OmePS AOs, respectively (Fig.4D), suggesting MOE25(PS) is more effective than MOE25(PO) and 2′OmePS AOs in inducing exon skipping and dystrophin restoration *in vivo*.

## Discussion

AO-mediated exon skipping therapeutics for DMD has garnered significant interest in the past decade, not solely for the potential benefits to patients with a devastating muscle-wasting disease (2,5), but also as a model system for the development of other AO-based therapeutics. Numerous studies have utilized AOs of different chemistries to modulate the pre-messenger RNA splicing of dystrophin. The AO chemistry of 2′-O-methoxyethyl phosphorothioate RNA has been extensively tested in various disease models with an appreciable safety profile [23], [33], [34], [35], [36], [37], while its potential in inducing exon-skipping in DMD remains to be established. Here we explored the potential of MOE AOs for exon-skipping in the murine dystrophin gene. Our results demonstrated that MOE AOs could induce effective exon-skipping *in vitro* (Fig. 1) and in local intramuscular studies (Fig. 4) with a PS backbone showing superiority to the ones with a PO backbone in inducing dystrophin exon-skipping. Of particular significance, 25-mer MOE PS AOs induced significantly higher levels of dystrophin expression in a local intramuscular study, thereby indicating that the MOE PS chemistry has promising potential for efficient exon-skipping of dystrophin in DMD.

As MOE AOs are highly similar to 2′OmePS in structure and in chemical properties and the latter is currently in clinical trials, we wanted to test whether MOE PS AOs have any advantage over the 2′OmePS chemistry. Therefore we directly compared MOE PS and 2′OmePS AOs *in vitro* and *in vivo* using a single intramuscular injection. RT-PCR data show that there was a significant increase in exon skipping efficiency in H2K *mdx* cells treated with MOE AOs compared to 2′OmePS, with the exception of MOE20(PS) (Fig. 3). Interestingly, although MOE PS AOs bear similar chemical structure to 2′OmePS, the former acts in a length-dependent manner as demonstrated in our current study and the latter showed less exon skipping activity with increased length as reported [32]. In a local intramuscular study, a significantly higher number of dystrophin-positive fibres was achieved with MOE25(PS) AOs compared with 2′OmePS (Fig. 4B), which was corroborated by RT-PCR and western blot with up to 20% of normal level of dystrophin protein detected in TA muscles treated with MOE25(PS) AOs (Fig. 4C–D). These data further indicate the potential of MOE PS AOs as an option in DMD therapy.

Although cellular uptake *in vitro* was measured in the presence of lipofectin, which did not reflect actual uptake efficiencies of the different chemistries *in vivo*, good correlation between cellular uptake of fluorescence-tagged MOE25(PS) and 2′Ome PS AOs and exon-skipping efficiency suggested that exon-skipping activity of both chemistries was similar in H2K *mdx* cells, and that the differences in apparent exon-skipping efficiency can be partly attributed to different efficiency in cellular uptake. Extrapolation of this result suggests that the differences in the number of positive fibres after intramuscular injection arose probably primarily from the difference in uptake efficiency, which in turn suggest that MOE25 PS is taken up more readily by cells after injection. However our cellular localization data with fluorescence-tagged MOE25(PS) and 2′OmePS in H2K *mdx* cells using confocal microscopy showed AOs localized much less in nuclei and a substantial amounts of labeled AOs were trapped in the cytoplasm (data not shown).

In conclusion, our study shows that MOE PS AOs could effectively induce exon skipping and dystrophin expression *in vitro* and in local intramuscular studies, demonstrating their potential as an alternative AO option for the treatment of DMD. MOE25 PS may be superior to 2′OMe PS chemistry in cellular uptake *in vivo* as shown indirectly via cellular uptake studies. Further studies will be required to determine the systemic efficacy of MOE25(PS) AOs in directing exon skipping and restoring dystrophin expression in *mdx* mice.

## Supporting Information

Figure S1 **Comparison between MOE and 2′OmePS AOs in inducing exon skipping in differentiated H_2_K** ***mdx*** **myotubes.** (A) RT-PCR results for 500 nM MOE and 2′OmePS AOs in differentiated H_2_K *mdx* myotubes at 48 h after transfection. (B) Quantification of percentage of exon 23 skipping for MOE and 2′OmePS AOs at 48 h after transfection in H2K *mdx* myotubes, showing the same pattern as detected in undifferentiated H2K *mdx* myoblasts.
(TIF)
