# Supplementary figures and images for: Effective Exon Skipping and Dystrophin Restoration by 2′-O-Methoxyethyl Antisense Oligonucleotide in Dystrophin-Deficient Mice
Source: PLoS One. 2013 Apr 26;8(4):e61584. doi: 10.1371/journal.pone.0061584 (PMC3637291; doi:10.1371/journal.pone.0061584)

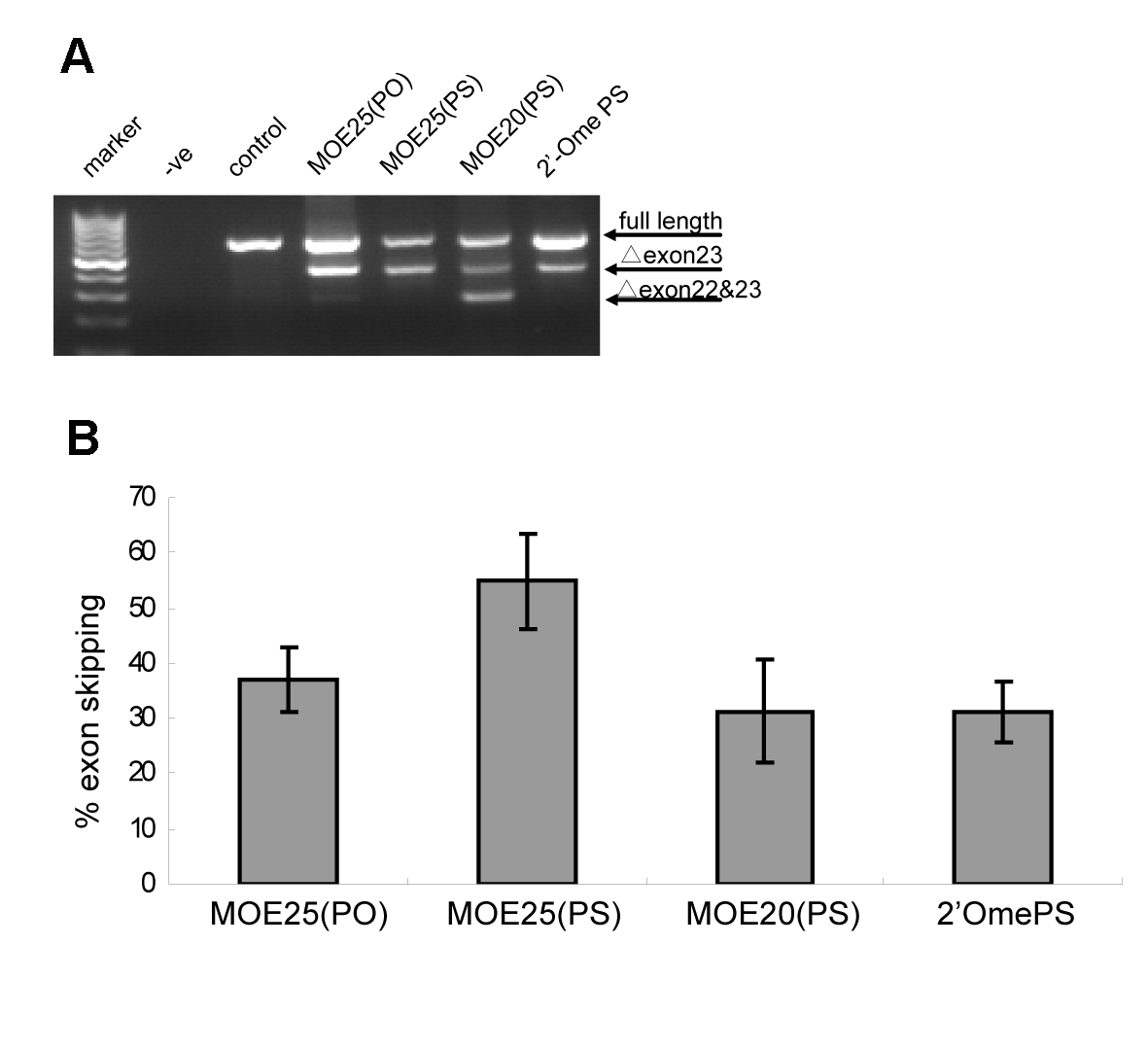

Supplement: Figure S1 — Comparison between MOE and 2′OmePS AOs in inducing exon skipping in differentiated H2K mdx myotubes. (A) RT-PCR results for 500 nM MOE and 2′OmePS AOs in differentiated H2K mdx myotubes at 48 h after transfection. (B) Quantification of percentage of exon 23 skipping for MOE and 2′OmePS AOs at 48 h after transfection in H2K mdx myotubes, showing the same pattern as detected in undifferentiated H2K mdx myoblasts. (TIF) [file pone.0061584.s001.tif]
